# Association Between Fasting Blood Glucose and Myocardial Infarction Risk: Findings From the 2015–2018 NHANES Database and Mendelian Randomization Studies

**DOI:** 10.1155/crp/6984101

**Published:** 2026-04-20

**Authors:** Bo Peng, Jianheng Liang, Lechong Yuan, Donglin Lin, Xiaoping Fan

**Affiliations:** ^1^ Department of Cardiovascular Surgery, Guangdong Provincial Hospital of Chinese Medicine, the Second Affiliated Hospital of Guangzhou University of Chinese Medicine, the Second Clinical College of Guangzhou University of Chinese Medicine, Guangzhou, 510120, Guangdong, China, gzucm.edu.cn; ^2^ Guangdong Provincial Key Laboratory of TCM Emergency Research, Guangzhou, 510120, Guangdong, China; ^3^ Department of General Surgery, Nan Fang Hospital, Southern Medical University, Guangzhou, 510515, China, fimmu.com; ^4^ Department of Cardiology, Zhu Jiang Hospital, Southern Medical University, Guangzhou, 510260, China, fimmu.com

**Keywords:** fasting blood glucose, Mendelian randomization, myocardial infarction, National Health and Nutrition Examination Survey

## Abstract

**Introduction:**

Fasting blood glucose and myocardial infarction share some common pathophysiological risk factors, but the exact relationship between them remains unclear. This study aims to provide evidence for the association between fasting blood glucose and myocardial infarction by analyzing data from the National Health and Nutrition Examination Survey (NHANES) 2015–2018 and Mendelian randomization (MR) analysis.

**Methods:**

A two‐sample MR study was conducted to explore the causal relationship between fasting blood glucose and myocardial infarction using summary statistics from genome‐wide association studies (GWAS). The inverse variance weighted (IVW) method and other supplementary MR methods were mainly used to verify the causal association, and sensitivity analysis was performed to confirm the robustness of the results. In addition, weighted multivariable adjusted logistic regression analysis was used to evaluate the relationship between fasting blood glucose and the myocardial infarction–related multivariable association model constructed with HDL as the core indicator based on NHANES data from 2015 to 2018.

**Results:**

A total of 4807 participants were included in the observational study based on NHANES data. Weighted multivariable adjusted logistic regression analysis showed a positive correlation between fasting blood glucose and the myocardial infarction model, with an odds ratio (OR) of −0.027 and a 95% confidence interval (CI) of [−0.042, −0.011]. MR analysis also indicated a causal relationship between myocardial infarction and fasting blood glucose (IVW: OR = 1.0026, 95% CI = 1.0006–1.0046, *p* = 0.0098). Sensitivity analysis further confirmed the robustness and reliability of these study results (all *p* > 0.05).

**Conclusion:**

There is a causal relationship between fasting blood glucose and myocardial infarction.

## 1. Introduction

Myocardial infarction is a severe disease caused by coronary heart disease, usually triggered by myocardial ischemia due to coronary artery stenosis or occlusion [1]. In the United States, the incidence of acute myocardial infarction is 261 per 100,000 population in men and 176 per 100,000 population in women [2]. In South Korea, the prevalence of myocardial infarction among adults aged 30 years and above has been increasing year by year [3]. Myocardial infarction has various risk factors, including age, smoking, hypertension, hypercholesterolemia, obesity, and diabetes mellitus [4], and can induce serious complications such as heart failure [5] and atrial fibrillation [6]. Among them, diabetes mellitus is becoming one of the most important factors inducing myocardial infarction. Compared with nondiabetic patients, the mortality rate of cardiovascular diseases increases more than twice in men and more than once in women [7]. The most critical factor of diabetes mellitus leading to myocardial infarction is hyperglycemia [8, 9]. Hyperglycemia can increase the conversion of aldose reductase substrates, and its overexpression will accelerate the occurrence of acute myocardial infarction related to atherosclerosis [10]. Hyperglycemia can also activate *β*, *δ*, and *θ* subtypes of protein kinase C (PKC). In mice, activation of PKCβ inhibits the transcription of arterial interleukin‐18 binding protein, leading to increased plaque formation, elevated cholesterol ester content, and increased macrophage infiltration [11]. Hyperglycemia can induce excessive production of reactive oxygen species (ROS), amplifying its damaging effect on endothelial cells [12].

These studies have clearly confirmed the close association between hyperglycemia and myocardial infarction in diabetic patients. However, studies on the relationship between fasting blood glucose levels and the etiology of myocardial infarction in the general population are still limited. Mendelian randomization (MR) is a commonly used research method in the field of bioinformatics to explore the genetic association between myocardial infarction and various exposure factors. This study also adopts a two‐sample MR method combined with genome‐wide association study (GWAS) data to explore the potential relationship between fasting blood glucose and myocardial infarction.

At the same time, based on the monitoring data of the National Health and Nutrition Examination Survey (NHANES) from 2015 to 2018, we conducted a large‐scale cross‐sectional study to explore the potential association between fasting blood glucose levels and myocardial infarction.

## 2. Materials and Methods

### 2.1. MR

#### 2.1.1. Study Design

Two‐sample MR is a commonly used randomization method in the field of bioinformatics for processing data from two groups of samples in a study, which can ensure the randomness of grouping and the reliability of experimental results. This method involves randomization of sample grouping and treatment allocation to minimize nonrandom factors in the experimental design and implementation process. By randomly assigning experimental groups and control groups, the relative fairness and balance of the two groups of samples under experimental conditions are ensured. The randomization principle can reduce the bias and error of experimental data and ensure the objectivity and scientificity of the results. This study uses data from the GWAS database to reveal the causal relationship between exposure factors and primary outcomes [12].

**FIGURE 1:**
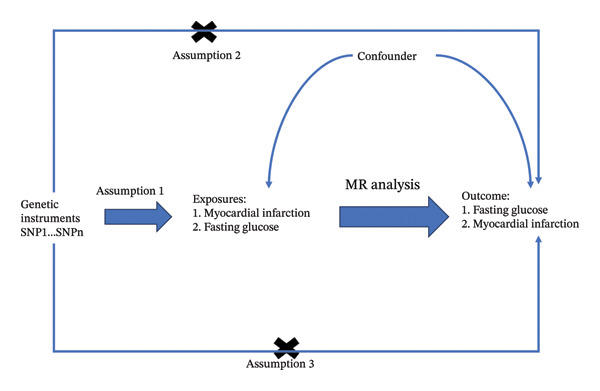
Design of a two‐sample Mendelian randomization study for myocardial infarction (MI) and fasting glucose.

The MR in this study is based on three assumptions: (1) Relevance assumption: The selected independent variables are directly related to the exposure factors; (2) independence assumption: The selected independent variables are independent of confounding variables; (3) exclusion restriction assumption: The selected independent variables should not directly affect the outcome unless through association with the exposure factors [13, 14] (Figure 1).

Four sets of GWAS data were selected in this study to explore the genetic significance of single nucleotide polymorphisms (SNPs) between myocardial infarction and fasting blood glucose.

#### 2.1.2. Data Sources

Four sets of summary data were obtained from the GWAS database for bidirectional MR analysis in this study. The data sets related to myocardial infarction are ukb‐b‐16662 and ukb‐e‐I21_CSA, including 463,010 and 8876 European participants, respectively; the data sets related to fasting blood glucose are ieu‐b‐113 and ebi‐a‐GCST90002234, including 13,310 and 9343 European participants, respectively. The diagnosis of myocardial infarction and fasting blood glucose in patients was based on the classification criteria of the American Heart Association and the American College of Physicians. The detailed study design and data control process have been reported in other literature types [14].

#### 2.1.3. Statistical Analysis

In this study, R software (Version 4.2.1) was used for data analysis through the TwoSampleMR package (Version 0.5.6) and the MRPRESSO package (Version 1.0). To avoid linkage disequilibrium, the criteria of kb = 10,000 and *r* ^2^ = 0.001 were adopted when aggregating SNPs. Meanwhile, *p* < 5 × 10^−8^ was set as the genome‐wide significance threshold to screen SNPs strongly associated with myocardial infarction and fasting blood glucose. Palindromic SNPs were excluded (because their direction of action in myocardial infarction and fasting blood glucose cannot be determined). Finally, the variance proportion of exposure was calculated using the *r* ^2^ value of each SNP, and the strength of instrumental variables was evaluated by the F statistic to avoid weak instrumental variable bias.

The main MR analysis adopted the classic inverse variance weighted (IVW) model method, which weights the effect of each genetic variant by the reciprocal of its standard error (or reciprocal of variance) to estimate the overall effect; at the same time, it reduces the estimation bias caused by heterogeneity through relative weight allocation and integrates the effect size estimates of multiple loci [15].

Four other statistical methods were also applied in this study, including weighted median estimation model (WME), weighted model‐based method (WM), MR‐Egger regression model (MER), and simple mode (SE). WME calculates the median of locus effect sizes and weights them according to their standard errors to handle outliers, providing a comprehensive estimate of the combined effect size of multiple genetic variants [16]; WM calculates the median of effect sizes of multiple genetic variants and combines them through weighted average to obtain the estimated value of the overall effect; MER combines the idea of Egger regression to evaluate the bias and symmetry of causal effect estimation, which can be used to evaluate the stability and consistency of estimated values; SE extracts basic genetic information and association patterns, and intuitively presents basic genetic characteristics such as genotype frequency distribution and genotype‐phenotype relationship.

The Harmonize tool was used to exclude SNPs with incompatible alleles and palindromic SNPs with minor allele frequency. Considering that there are differences in SNPs extracted from different experimental environments and experimental designs, bidirectional MR analysis may have heterogeneity, leading to errors in the calculated causal relationship. Therefore, heterogeneity test and MERregression test were performed on the main IVW analysis method, and the *p* values were 0.1957 and 0.2183, respectively, indicating no heterogeneity.

Horizontal pleiotropy in MR analysis was evaluated by the intercept value in MR‐Egger, and the existence of pleiotropy was analyzed by the *p* value of the heterogeneity test. If *p* > 0.05, pleiotropy in causal analysis can be ignored. Finally, leave‐one‐out analysis was used to test the consistency of the results.

### 2.2. NHANES

#### 2.2.1. Study Population

**FIGURE 2:**
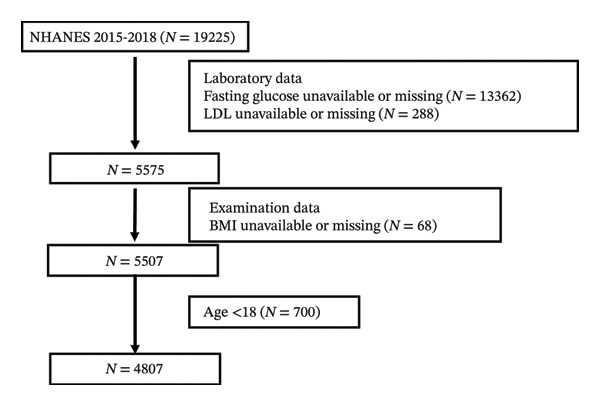
Flowchart of participants in NHANES from 2015 to 2018. BMI, body mass index; HDL, high‐density lipoprotein; LDL, low‐density lipoprotein.

The data used in this study are publicly available from the NHANES database (https://www.cdc.gov/nchs/nhanes/index.htm). The research data of NHANES have been approved by the Research Ethics Review Board of the National Center for Health Statistics (NCHS), and all participants have signed informed consent forms. The data used in this study are de‐identified public data, which have been approved by the Institutional Review Board. This analysis uses data from two cycles of NHANES (2015–2016 and 2017–2018) to accumulate an appropriate sample size through independent samples. Participants with missing key variables or under 18 years of age were excluded (Figure 2).

#### 2.2.2. Definition of Exposure and Outcome

Fasting blood glucose was used as the exposure factor, and hemophiliacs, patients excluded for chemotherapy safety, volunteers with fasting time less than 9 h, and those who refused blood collection, were excluded. Glucose in plasma is converted to glucose‐6‐phosphate (G‐6‐P) by hexokinase in the presence of adenosine triphosphate (ATP); subsequently, in the presence of nicotinamide adenine dinucleotide phosphate (NADP^+^), G‐6‐P is converted to glucose‐6‐phosphate dehydrogenase by NADP^+^. The glucose concentration was reflected by measuring the increase in absorbance at 340 nm (caused by the reduction of NADP^+^ to nicotinamide adenine dinucleotide phosphate hydrogen [NADPH]).

High‐density lipoprotein (HDL), a marker closely related to myocardial infarction, was used as the outcome indicator to construct a myocardial infarction–related model. Serum collection of HDL followed the guidelines issued by the American Heart Association and the American College of Cardiology. After processing and storage, serum samples were transported to the University of Minnesota in Minneapolis for analysis. Samples were stored in vials under appropriate freezing conditions (−30°C) until transported to the University of Minnesota for testing.

Given the unavailability of direct clinical diagnosis data, hospitalization records, and mortality data related to myocardial infarction in the 2015–2018 NHANES database, we constructed a myocardial infarction–related multivariable association model with HDL as the core outcome indicator in this study. HDL is a well‐recognized vascular protective factor and an established independent inverse predictor of myocardial infarction and coronary heart disease in numerous epidemiological and clinical studies, with its serum level closely and stably associated with the occurrence and development of myocardial infarction. On this basis, we further incorporated a variety of myocardial infarction–related clinical covariates (including blood pressure, blood lipid indicators, and metabolic comorbidities) into the model for multivariable adjustment, which can effectively improve the specificity and reliability of the model for reflecting the risk of myocardial infarction. This indirect evaluation method based on core biological markers and comprehensive covariates is a commonly used alternative strategy in population‐based cross‐sectional studies when direct disease outcome data are lacking, and has been verified and applied in previous cardiovascular disease‐related studies based on the NHANES database.

#### 2.2.3. Other Covariates Used in NHANES

Based on existing literature and data, this study collected potential covariates related to myocardial infarction and cardiovascular events, including gender (male, female), race/ethnicity (Mexican American, other races, non‐Hispanic white, non‐Hispanic black), poverty‐to‐income ratio (PIR; < 1.2 or ≥ 1.2), sleep disorders (yes, no, unknown), thoracalgia (yes, no, unknown), hypertension (yes, no, unknown), hypercholesterolemia (yes, no, unknown), systolic blood pressure (SBP) and diastolic blood pressure (DBP) (mmHg), triglycerides (TGs) (mg/dL), and low‐density lipoprotein (LDL, mg/dL). SBP and DBP followed the guidelines of the American Heart Association, taking the average of three resting measurements; TGs (mg/dL) were detected in serum through a series of coupled reactions.

#### 2.2.4. Data Analysis

For NHANES data analysis, multivariable adjusted logistic regression was used to evaluate the relationship between HDL, LDL, and fasting blood glucose. After covariate adjustment, three models were evaluated: Model 1 (unadjusted); Model 2 (adjusted for gender, age and race/ethnicity); Model 3 (on the basis of Model 2, further adjusted for age, gender, race/ethnicity, PIR, LDL, TGs, sleep disorders, thoracalgia, hypertension, hyperlipidemia, SBP, and DBP). Results are expressed as odds ratio (OR) or *β* coefficient (95% confidence interval [CI]). Considering the complex probability cluster design of NHANES, weight factors were included in the statistical analysis, and curve fitting was performed [17].

## 3. Results

### 3.1. MR Analysis Results

A bidirectional MR design was adopted in this study to explore the effect of fasting blood glucose on myocardial infarction and the reverse effect of myocardial infarction on fasting blood glucose, respectively, and various statistical methods were used to verify the robustness of the association.

**FIGURE 3:**
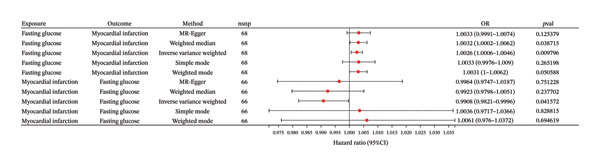
The forest plot demonstrates the relationship between myocardial infarction and fasting glucose in Mendelian randomization (MR) analysis.

In the analysis with fasting blood glucose as the exposure factor and myocardial infarction as the outcome factor, a total of 68 eligible SNP loci were screened out. The results of different MR analysis methods showed that IVW analysis indicated a significant positive causal relationship between fasting blood glucose and myocardial infarction (OR = 1.0026, 95% CI = 1.0006–1.0046, *p* = 0.0098); the result of weighted median method was consistent with IVW method and statistically significant (OR = 1.0032, 95% CI = 1.0002–1.0062, *p* = 0.0387); MER (OR = 1.0033, 95% CI = 0.9991–1.0074, *p* = 0.1254), SE (OR = 1.0033, 95% CI = 0.9976–1.0090, *p* = 0.2652) and weighted mode (OR = 1.0031, 95% CI = 1.0000–1.0062, *p* = 0.0506) did not reach statistical significance, but the direction of effect was consistent with the core results, further supporting that elevated fasting blood glucose may increase the risk of myocardial infarction (Figure 3).

In the reverse analysis with myocardial infarction as the exposure factor and fasting blood glucose as the outcome factor, a total of 75 valid SNP loci were extracted. IVW analysis showed that myocardial infarction had a significant effect on fasting blood glucose (OR = 0.9908, 95% CI = 0.9821–0.9996, *p* = 0.0416); among other analysis methods, the direction of effect of weighted median method (OR = 0.9923, 95% CI = 0.9798–1.0051, *p* = 0.2377), MERregression (OR = 0.9964, 95% CI = 0.9747–1.0187, *p* = 0.7512), SE (OR = 1.0036, 95% CI = 0.9717–1.0366, *p* = 0.8288), and weighted mode (OR = 1.0061, 95% CI = 0.9760–1.0372, *p* = 0.6496) was consistent with IVW method, but did not reach statistical significance, suggesting that there may be a bidirectional causal association between the two (Figure 3). All MR analysis results are summarized in Table S1.

Heterogeneity test results showed that in the Cochran *Q* test, the *Q* value of the analysis of fasting blood glucose on myocardial infarction was 57.38 (*Q*_df = 65, *Q*_pval = 0.74), and the *Q* value of the analysis of myocardial infarction on fasting blood glucose was 65.50 (*Q*_df = 67, *Q*_pval = 0.52), both greater than 0.05, indicating that there was no significant heterogeneity in both analyses. MER intercept test was used to evaluate horizontal pleiotropy, and the results showed that the intercept was −1.744*e* − 05 (*p* = 0.726) when fasting blood glucose was used as the exposure factor, and −0.0039 (*p* = 0.589) when myocardial infarction was used as the exposure factor, both greater than 0.05, suggesting no obvious horizontal pleiotropy (Table 1).

**TABLE 1:** Heterogeneity of directional pleiotropy and the MR‐Egger test for directional pleiotropy.

Outcome	Exposure	*Q*	*Q*_df	*Q*_*p*val
*Heterogeneity*				
Myocardial infarction	Fasting glucose	57.38	65	0.74
Fasting glucose	Myocardial infarction	65.50	67	0.52

**Outcome**	**Exposure**	**Intercept**	**SE**	***p*val**

*MR-Egger pleiotropy test*				
Fasting glucose	Myocardial infarction	−1.744*e* − 05	4.965*e* − 05	0.726
Myocardial infarction	Fasting glucose	−0.0039	0.0056	0.589

The forest plot of the causal relationship between myocardial infarction and fasting blood glucose predicted by genetic analysis is shown in Figure 4, which clearly presents the distribution of effect sizes of different SNP loci; the scatter plot of SNP effect sizes between myocardial infarction and fasting blood glucose is shown in Figure 5, which intuitively reflects the association strength between genetic variants and exposure factors, outcome indicators. In addition, R^2^ and F statistics were calculated by effective allele frequency (EAF) and effect estimate (BETA) to evaluate the strength of instrumental variables. The results showed that all F statistic values were greater than 10, indicating that the instrumental variables were sufficiently strong to effectively avoid weak instrumental variable bias.

FIGURE 4Forest plot of the causal effects of single nucleotide polymorphisms (SNPs). (a) Causal effects of SNPs on fasting glucose as the exposure and myocardial infarction as the outcome. (b) Causal effects of SNPs on myocardial infarction as the exposure and fasting glucose as the outcome.

**Figure:**
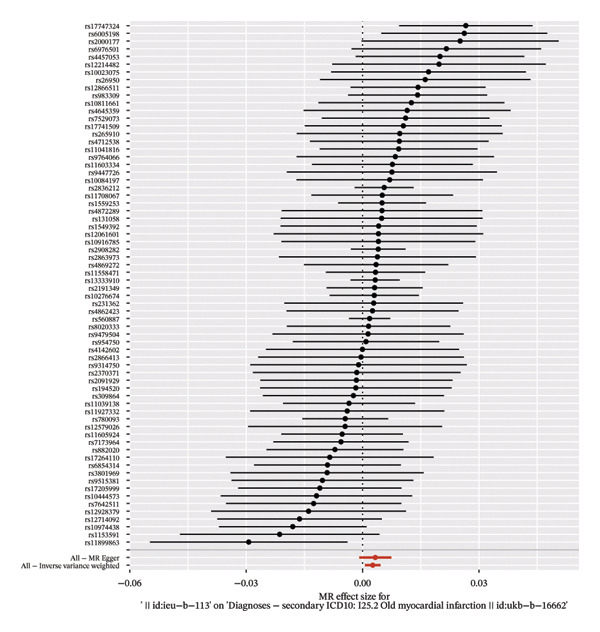
(a)

**Figure:**
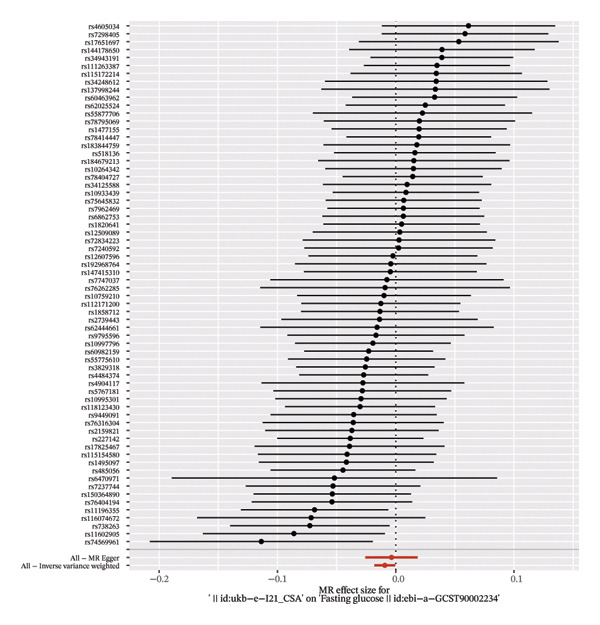
(b)

FIGURE 5Scatter plots of the genetic associations between myocardial infarction and fasting glucose. (a) Scatter plot of the genetic association between fasting glucose as the exposure and myocardial infarction as the outcome. (b) Scatter plot of the genetic association between myocardial infarction as the exposure and fasting glucose as the outcome.

**Figure:**
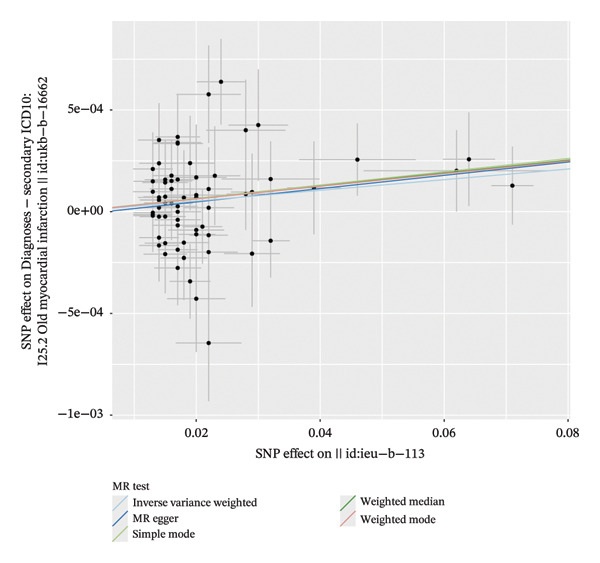
(a)

**Figure:**
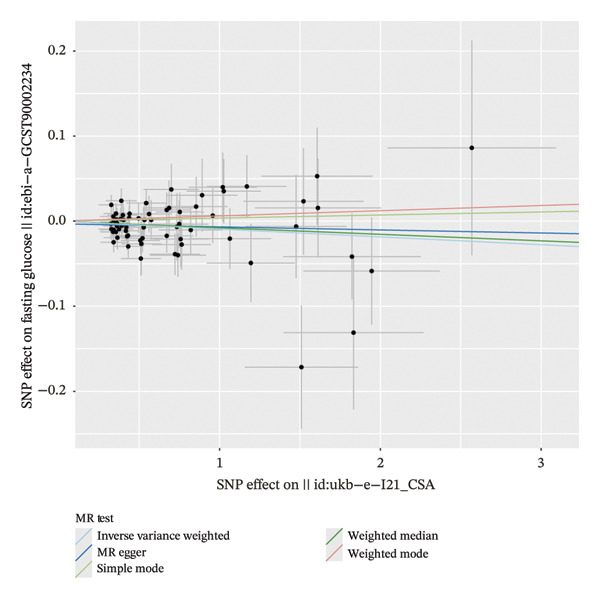
(b)

### 3.2. NHANES Baseline Characteristics and Study Results

A total of 4807 participants were included in this analysis, with males accounting for 48.16% (weighted proportion). The 1st–3rd quartile ranges of fasting blood glucose were 21–97, 97–108, and 108–479, respectively. Overall, with the increase of fasting blood glucose quartiles, the weighted average HDL level gradually decreased (low: 59.8 ± 17.8; medium: 55.0 ± 15.6; high: 51.4 ± 17.3, *p* < 0.0001). There were statistically significant differences in LDL, age, body mass index (BMI), SBP, DBP, TG, gender, race, hypertension, hypercholesterolemia, and sleep disorders among the study population (all *p* < 0.05) (Table 2).

**TABLE 2:** Baseline characteristics of the participants, weighted.

Characteristic	Fasting glucose			*p* value
	Low	Middle	High	
Age	40.5 ± 16.8	46.8 ± 16.8	56.1 ± 15.6	< 0.0001
PIR	2.8 ± 1.7	2.9 ± 1.6	2.9 ± 1.7	0.0946
BMI	27.2 ± 6.6	28.9 ± 6.5	32.0 ± 7.6	< 0.0001
SBP	117.1 ± 15.8	122.1 ± 15.7	128.6 ± 18.0	< 0.0001
DBP	68.5 ± 11.4	70.9 ± 11.9	71.7 ± 13.9	< 0.0001
HDL	59.8 ± 17.8	55.0 ± 15.6	51.4 ± 17.3	< 0.0001
TG	88.9 ± 53.9	104.6 ± 62.4	127.7 ± 67.6	< 0.0001
LDL	108.4 ± 33.8	114.3 ± 34.8	111.2 ± 37.6	< 0.0001
Gender				< 0.0001
Male	36.5	53.8	55.8	
Female	63.5	46.2	44.2	
RACE				< 0.0001
Mexican American	8.2	8.7	10.4	
Other Hispanic	7.1	6.5	6.4	
Non‐Hispanic White	60.5	64.8	65.2	
Non‐Hispanic Black	15.2	9	8.7	
Other race—including multiracial	9.1	11	9.3	
Hypertension				< 0.0001
Yes	19.1	28.5	49.9	
No	80.9	71.3	50.1	
Hypercholesteremia				< 0.0001
Yes	21.6	30.9	46.6	
No	78.2	69	52.7	
Sleep disorders				< 0.0001
Yes	26.1	30.4	35.3	
No	73.9	69.6	64.7	
Thoracalgia				0.2871
Yes	30.8	28.9	30.3	
No	69.1	71.1	69.4	

*Note:* Mean ± standard error (SE) for continuous variables and percentage (%) for categorical variables.

Abbreviations: BMI, body mass index; DBP, diastolic blood pressure; HDL, high‐density lipoprotein; LDL, low‐density lipoprotein; PIR, poverty‐to‐income ratio; SBP, systolic blood pressure; TG, triglyceride.

In the unadjusted model, fasting blood glucose was negatively correlated with HDL (*β* = −0.096, 95% CI = −0.111 to −0.081, *p* < 0.001). After adjusting for covariates in Model 1 (*β* = −0.099, 95% CI = −0.114 to −0.084, *p* < 0.001) and Model 2 (adjusted for myocardial infarction, *β* = −0.027, 95% CI = −0.042 to −0.011, *p* = 0.0011), this significant correlation still existed. When fasting blood glucose was converted from a continuous variable to a categorical variable (tertile), the HDL of participants with lower fasting blood glucose was 3.037 mg/dL lower than that of participants with higher fasting blood glucose(Table 3).

**TABLE 3:** Results of the weighted multiple logistic regression analysis for HDL and fasting glucose.

Exposure	Nonadjusted (95% CI)	Adjust I (95% CI)	Adjust II (95% CI)
GLU	−0.096 (−0.111, −0.081) < 0.00001	−0.099 (−0.114, −0.084) < 0.00001	−0.027 (−0.042, −0.011) 0.00110

*GLU tripartite group*			
Low	Reference	Reference	Reference
Middle	−4.745 (−5.908, −3.582) < 0.00001	−4.070 (−5.190, −2.949) < 0.00001	−2.204 (−3.665, −0.743) 0.00314
High	−8.408 (−9.597, −7.218) < 0.00001	−9.074 (−10.294, −7.853) < 0.00001	−3.037 (−4.554, −1.519) 0.00009

*Note:* Model 1 (nonadjusted): no covariates adjusted. Model 2 (Adjust I): adjusted for age, gender, and race/ethnicity. Model 3 (Adjust II): adjusted for age, gender, race/ethnicity, poverty‐to‐income ratio (PIR), systolic blood pressure, diastolic blood pressure, triglycerides (TG), hypertension, hypercholesterolemia, body mass index (BMI), sleep disorders, thoracalgia, and low‐density lipoprotein (LDL). The effect size is expressed as a *β* coefficient; *p* values are indicated after each effect estimate.

Abbreviations: 95% CI, 95% confidence interval; GLU, fasting glucose; HDL, high‐density lipoprotein; OR, odds ratio.

**FIGURE 6:**
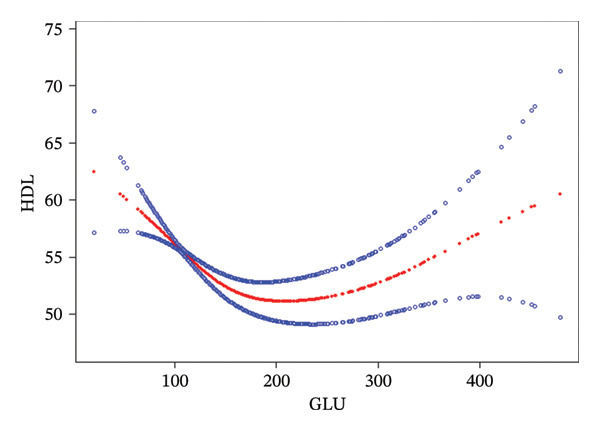
The probability association between HDL and fasting glucose. The adjusted model for potential confounders includes age, gender, race, PIR (previously mentioned), systolic blood pressure, diastolic blood pressure, triglycerides, hypertension, hypercholesterolemia, body mass index, sleep disorders, thoracalgia, and LDL. The red curve represents the fitted smooth curve, while the blue lines indicate the 95% confidence intervals.

To clarify the nonlinear characteristics of the relationship between fasting blood glucose and the myocardial infarction model constructed with HDL as the main exposure, the statistical significance of the threshold effect was further verified by the likelihood ratio test. The results showed that the likelihood ratio statistic was 7.03, *p* = 0.0082, indicating that the two‐piece piecewise linear model had a better fitting effect than the single linear model (adjusted *R* ^2^ increased from 0.013 to 0.021), confirming that there was a significant threshold effect in the relationship between fasting blood glucose and the myocardial infarction model constructed with HDL as the main exposure (threshold value was 163 mg/dL). For the interval of fasting blood glucose < 163 mg/dL, the effect estimate was −0.027 (95% CI: −0.043∼‐0.011), *p* = 0.0010, and the corresponding F statistic was 7.56 (*p* < 0.01), suggesting that the negative correlation between fasting blood glucose and the myocardial infarction model constructed with HDL as the main exposure in this interval had statistical robustness; for the interval of fasting blood glucose ≥ 163 mg/dL, the effect estimate was −0.099 (95% CI: −0.133∼−0.064), *p* < 0.0001, and the F statistic was 28.34 (*p* < 0.0001), indicating that the strength of the negative correlation between the two in this interval was significantly enhanced, and the statistical reliability of the association was higher; the effect difference test between the two intervals showed that the difference value was −0.072 (95% CI: −0.115∼−0.029), *p* = 0.0012, confirming that after fasting blood glucose exceeded the threshold of 163 mg/dL, the negative impact on HDL was significantly increased (Table 4, Figure 6).

**TABLE 4:** Analysis of threshold effects of fasting glucose on HDL.

Analysis model	Inflection point (mg/dL)	Effect estimate (95% CI)	*p* value	Adjusted *R* ^2^	*F* statistic
Single linear model	—	0.028 (−0.000, 0.056)	0.0520	0.013	3.89
Two‐piece piecewise linear model (≤ threshold)	163	−0.027 (−0.043, −0.011)	0.0010	0.021	7.56
Two‐piece piecewise linear model (> threshold)	163	−0.099 (−0.133, −0.064)	< 0.0001	0.021	28.34
Threshold effect test (LRT)	—	—	0.0082	—	—

*Note:* All models were adjusted for age, gender, race/ethnicity, poverty‐to‐income ratio (PIR), systolic blood pressure, diastolic blood pressure, triglycerides (TG), hypertension, hypercholesterolemia, body mass index (BMI), sleep disorders, thoracalgia, and low‐density lipoprotein (LDL). The effect size is expressed as a *β* coefficient, representing the change in HDL (mg/dL) per 1 mg/dL increase in fasting glucose. The likelihood ratio test compares the fit of the single linear model versus the two‐fold piecewise linear model; *p* < 0.05 indicates a significant threshold effect.

Abbreviations: 95% CI, 95% confidence interval; HDL, high‐density lipoprotein.

The adjusted *R* ^2^ of the single linear model was only 0.013, indicating that its ability to explain the relationship between fasting blood glucose and HDL was limited, while the adjusted *R* ^2^ of the two‐piece piecewise linear model increased to 0.021, and the overall *F* statistic was 15.87 (*p* < 0.0001), indicating that after introducing the threshold, the model could more fully explain the nonlinear association between the two, further supporting the authenticity of the threshold effect.

To evaluate whether the correlation between HDL and fasting blood glucose was consistent in the overall population and different subgroups, subgroup analysis was performed. Stratified interaction analysis was conducted based on gender, race, age, hypertension, hypercholesterolemia, sleep disorders, and thoracalgia. The results showed that there was no significant interaction among HDL and gender, race, age, hypertension, hypercholesterolemia, sleep disorders, or thoracalgia (interaction *p* > 0.05). This indicates that the negative correlation between HDL and fasting blood glucose is robust in populations with different demographic characteristics, socioeconomic status, and disease states and may be applicable to various populations (Table 5).

**TABLE 5:** Subgroup analysis of the association between fasting glucose and myocardial infarction.

	OR (95% CI)	*p* for interaction
Gender		0.128
Male	−0.015 (−0.036, 0.005) 0.1432	
Female	−0.040 (−0.065, −0.015) 0.0018	
Race		0.9036
Mexican American	−0.030 (−0.084, 0.023) 0.2631	
Other Hispanic	−0.008 (−0.058, 0.042) 0.7548	
Non‐Hispanic White	−0.032 (−0.054, −0.010) 0.0036	
Non‐Hispanic Black	−0.021 (−0.059, 0.018) 0.2956	
Other race—including multiracial	−0.015 (−0.079, 0.049) 0.6410	
Age		0.2742
< 35	−0.116 (−0.159, −0.073) < 0.0001	
35–50	−0.075 (−0.102, −0.047) < 0.0001	
> 50	−0.085 (−0.099, −0.070) < 0.0001	
Hypertension		0.2072
Yes	−0.018 (−0.039, 0.002) 0.0742	
No	−0.038 (−0.063, −0.014) 0.0023	
Don’t know	0.485 (−6.224, 7.193) 0.8874	
Hypercholesteremia		0.3899
Yes	−0.032 (−0.054, −0.011) 0.0032	
No	−0.022 (−0.045, 0.002) 0.0736	
Don’t know	0.156 (−0.147, 0.458) 0.3123	
Sleep disorders		0.4627
Yes	−0.034 (−0.058, −0.009) 0.0078	
No	−0.022 (−0.042, −0.001) 0.0405	
Don’t know	−0.019 (−0.370, 0.333) 0.9166	
Thoracalgia		0.817
Yes	−0.025 (−0.050, −0.000) 0.0493	
No	−0.029 (−0.050, −0.008) 0.0066	
Don’t know	0.953 (−4.027, 5.933) 0.7076	

*Note:* The results of the subgroup analysis were adjusted for all covariates: age, gender, race, PIR, LDL, triglycerides, sleep disability, thoracalgia, hypertension, hyperlipidemia, systolic blood pressure, diastolic blood pressure, systolic blood pressure, diastolic blood pressure.

## 4. Discussion

Through the dual verification of bidirectional MR analysis and NHANES cross‐sectional study, this study clearly confirmed the causal relationship between fasting blood glucose and myocardial infarction, and first revealed the positive impact of elevated fasting blood glucose on the risk of myocardial infarction in nondiabetic populations, providing new genetic and epidemiological evidence for understanding the pathophysiological association between the two. Due to the lack of direct myocardial infarction diagnosis data in NHANES, the HDL‐based multivariable association model was used to indirectly evaluate the myocardial infarction risk. The following is a critical interpretation and detailed elaboration on the core results of the study, in‐depth comparison with existing literature, advantages and limitations of the study, and contributions to the academic field and clinical practice.

### 4.1. Direct Interpretation of the Core Results of This Study

In the MR analysis, the IVW results with fasting blood glucose as the exposure factor showed (OR = 1.0026, 95% CI = 1.0006–1.0046, *p* = 0.0098) that for each unit increase in genetically predicted fasting blood glucose level, the risk of myocardial infarction increased significantly, while in the reverse MR analysis, the impact of myocardial infarction on fasting blood glucose was also statistically significant (IVW: OR = 0.9908, 95% CI = 0.9821–0.9996, *p* = 0.0416), suggesting that there may be a bidirectional causal association between the two, but the positive driving effect of fasting blood glucose on myocardial infarction is the core of this study. The weighted multivariable adjusted logistic regression analysis of NHANES data further verified this conclusion: After adjusting for age, gender, race, and metabolic‐related covariates, fasting blood glucose was still significantly negatively correlated with the myocardial infarction model (with HDL as the core marker) (*β* = −0.027, 95% CI = −0.042 to −0.011, *p* = 0.0011), and the HDL level of the group with the highest fasting blood glucose was 3.037 mg/dL lower than that of the group with the lowest fasting blood glucose, indirectly reflecting the increased risk of myocardial infarction.

It is worth noting that the smooth curve fitting analysis found that there was a threshold effect in the relationship between fasting blood glucose and HDL (inflection point was 163 mg/dL): Before the inflection point, the two were moderately negatively correlated; after the inflection point, the strength of the negative correlation was significantly enhanced (*β* = −0.099, 95% CI = −0.133 to −0.064, *p* < 0.0001). This finding suggests that when fasting blood glucose exceeds 163 mg/dL, its inhibitory effect on the vascular protective factor HDL is sharply enhanced, which may become a key turning point for the increased risk of myocardial infarction, providing a potential reference threshold for clinical risk stratification. In addition, subgroup analysis showed that this association was robust in populations with different genders, races, ages, and comorbidity statuses (interaction *p* > 0.05), indicating that the impact of fasting blood glucose on myocardial infarction has wide population applicability.

### 4.2. In‐Depth Comparison and Integration With Existing Literature

Previous studies have clearly confirmed that hyperglycemia in diabetic patients is closely associated with an increased risk of myocardial infarction [7–12]. For example, hyperglycemia can increase the risk of myocardial infarction through mechanisms such as activating the aldose reductase pathway to accelerate atherosclerosis [8], activating PKC subtypes to promote plaque formation [9], and inducing excessive production of ROS to damage endothelial cells [10–12]. The results of this study are consistent with the above conclusions, but further expand the evidence boundary: It is the first time to confirm through genetic epidemiological methods that even in nondiabetic populations, physiological elevation of fasting blood glucose (not meeting the diagnostic criteria for diabetes mellitus) can significantly increase the risk of myocardial infarction, breaking the traditional cognition that “only diabetic patients need to pay attention to blood glucose‐related cardiovascular risks” [18], supplementing the continuous evidence of the “blood glucose–myocardial infarction” association, and suggesting that the damage of blood glucose to the cardiovascular system is a gradual process rather than a “sudden” effect in the diabetic stage.

Atherosclerosis is the core pathological basis of myocardial infarction [19], and the results of this study form a logical closed loop with studies on atherosclerosis‐related mechanisms [20]. A clinical study based on ultrasound showed that for each 1 mg/dL increase in fasting blood glucose, the carotid intima–media thickness (IMT)—a key indicator of atherosclerosis—increased by about 0.24% [21]. This mechanism provides pathophysiological support for the “elevated fasting blood glucose ⟶ increased risk of myocardial infarction” observed in this study [22, 23]. In addition, the association between a SNP (rs48662423) of the acyl‐coenzyme A synthetase long‐chain family member 1 (ACSL1) gene and fasting blood glucose and atherosclerosis found in this study [24, 25] further verified the chain pathway of “elevated fasting blood glucose ⟶ progression of atherosclerosis ⟶ myocardial infarction” from the genetic level, closely linking population epidemiological evidence with molecular mechanism research.

Fasting blood glucose is a core component of the TG‐glucose (TyG) index [26], which has been confirmed to be closely associated with an increased risk of coronary heart disease [27], and coronary heart disease is an important prestage of myocardial infarction [28]. The results of this study support the mechanism of endothelial dysfunction mediated by the TyG index: elevated fasting blood glucose leads to an increase in the TyG index, which in turn impairs the L‐arginine/nitric oxide (NO) pathway [29], activates the renin–angiotensin system (RAS) [30], promotes the production of asymmetric dimethylarginine (ADMA) [31], reduces NO synthesis and bioavailability [32, 33], and ultimately exacerbates coronary artery constriction and endothelial damage, promoting the progression of atherosclerosis [34–38].

At the same time, the results of this study are complementary to studies related to thrombosis. Previous studies have confirmed that elevated fasting blood glucose can directly activate coagulation function [39], shorten the activated partial thromboplastin time [40], and increase the risk of venous thromboembolism [41], while thrombosis is the direct cause of myocardial infarction [42]. Through population‐level association analysis, this study verified the pathophysiological chain of “elevated fasting blood glucose ⟶ enhanced coagulation function ⟶ thrombosis ⟶ myocardial infarction,” further improving the mechanism network of fasting blood glucose affecting myocardial infarction.

The results of reverse MR analysis showed that myocardial infarction had a significant effect on fasting blood glucose (IVW: OR = 0.9908, 95% CI = 0.9821–0.9996, *p* = 0.0416), and the OR value was slightly lower than 1, suggesting that myocardial infarction may have a weak protective effect on fasting blood glucose levels; that is, the genetic susceptibility associated with myocardial infarction may be associated with a slight decrease in fasting blood glucose levels. The discovery of this two‐way causal association reflects the complex interactive regulation mechanism between metabolism and the cardiovascular system, and its potential biological pathway can be speculated from the following aspects.

From the perspective of pathophysiology, myocardial infarction, as a serious acute cardiovascular event, may affect blood glucose metabolism through long‐term adaptive adjustment of the body’s stress response. When an acute myocardial infarction occurs, the body starts a stress response. Sympathetic nerve excitement leads to a large release of catecholamines, which may promote liver glycogen decomposition and gluconeogenesis in the short term, causing transient hyperglycemia [43]. However, from the perspective of genetic susceptibility, reverse MR analysis reveals a long‐term potential association, and it is speculated that there may be a compensatory metabolic regulation mechanism: Individuals carrying myocardial infarction susceptibility genes may form a relatively low fasting blood glucose level through compensatory adaptation at the evolutionary level to reduce the damage of hyperglycemia to blood vessels, thus offsetting the genetic risk of myocardial infarction to a certain extent [44]. This “risk‐compensation” mechanism is not uncommon in the genetic association of complex diseases. For example, some cardiovascular disease susceptibility genes are also associated with the protective phenotype of lipid metabolism [45].

### 4.3. Explanation for Inconsistencies in Previous Studies

Some previous cross‐sectional studies did not observe a significant association between fasting blood glucose and myocardial infarction in nondiabetic populations [43], which is inconsistent with the results of this study. Through comparative analysis, we believe that the core reasons for the differences are as follows: ① Insufficient sample size: This study included 4807 participants, while some previous studies had only hundreds of samples, with limited statistical power, making it difficult to detect weak but significant associations; ② inadequate covariate adjustment: This study included more than 10 key covariates, such as gender, race, metabolic indicators, and comorbidities, while previous studies may not have adjusted for important confounding factors such as LDL and sleep disorders; ③ failure to identify threshold effects: Most previous studies used a single linear model and failed to find the nonlinear association between fasting blood glucose and myocardial infarction, while this study revealed a threshold of 163 mg/dL through a piecewise linear model, explaining the phenomenon of “no significant association at low blood glucose levels and significant association at high blood glucose levels.” This study effectively solved the limitations of previous studies through large samples, multiple covariate adjustments, and nonlinear analysis, and obtained more reliable conclusions.

### 4.4. Advantages and Limitations of the Study

This study innovatively combines MR analysis with a large‐scale cross‐sectional study. MR analysis uses the random allocation characteristics of genetic variants to establish the causal relationship between fasting blood glucose and myocardial infarction from a genetic perspective, effectively excluding reverse causality and confounding bias [13, 14]; the NHANES cross‐sectional study provides population‐level epidemiological evidence, reflecting the association pattern in the real world. The results of the two designs are mutually verified, significantly enhancing the reliability and external validity of the conclusions.

Rigorousness and comprehensiveness of statistical methods: MR analysis adopts a variety of complementary methods, such as IVW, weighted median, and MR‐Egger, and verifies the robustness of the results through Cochran *Q* test (heterogeneity *p* > 0.05), MERintercept test (horizontal pleiotropy *p* > 0.05) and leave‐one‐out analysis, ensuring the reliability of causal inference; NHANES analysis adopts multiple model covariate adjustments (unadjusted, partially adjusted, fully adjusted) and comprehensively explores the association pattern through smooth curve fitting, piecewise linear model, and subgroup analysis, improving the accuracy and depth of the results.

However, this study also has limitations. The GWAS data used in the MR analysis are all from European populations (myocardial infarction–related datasets: ukb‐b‐16662 includes 463,010 European participants, and ukb‐e‐I21_CSA includes 8876 European participants; fasting blood glucose‐related datasets: ieu‐b‐113 includes 13,310 European participants, and ebi‐a‐GCST90002234 includes 9343 European participants). There are differences in genetic background and lifestyle among different races, which may limit the extrapolation of the results to other races, such as Asians and Africans. Future MR analysis of multiethnic GWAS data is needed to verify the racial applicability of the conclusions. Although NHANES analysis included more than 10 covariates, there may still be unmeasured confounding factors, such as dietary habits (high‐sugar, high‐fat diet), exercise level, genetic background (other genetic variants except SNPs), and psychological factors, which may have a potential impact on the association between fasting blood glucose and myocardial infarction.

### 4.5. Academic Contributions of This Study to Existing Literature

This study is the first to clarify the bidirectional causal relationship between fasting blood glucose and myocardial infarction through bidirectional MR analysis, focusing on nondiabetic populations, filling the research gap of “causal association between high‐normal blood glucose range and myocardial infarction,” expanding the association between blood glucose and myocardial infarction from diabetic patients to the general population, and improving the continuous evidence chain of the association between the two. Previous studies mostly assumed a linear association between blood glucose and myocardial infarction, while this study found a threshold effect of 163 mg/dL through a piecewise linear model, providing a new perspective for understanding the complex association between the two, explaining the inconsistencies in previous studies, and providing a new analysis paradigm for subsequent related studies. This study combines the dual design of MR analysis and a large‐scale cross‐sectional study, uses a variety of statistical methods to verify the robustness of the results, and includes comprehensive covariate adjustment and subgroup analysis, providing a replicable and promotable research paradigm for exploring the causal relationship between metabolic indicators and cardiovascular diseases.

## 5. Conclusion

Through bidirectional MR analysis and NHANES analysis, this study confirmed the causal relationship between fasting blood glucose and myocardial infarction, indicating that elevated fasting blood glucose increases the risk of myocardial infarction in nondiabetic patients.

## Funding

This study was supported by National Natural Science Foundation of China (No. 82374240).

## Conflicts of Interest

The authors declare no conflicts of interest.

## Supporting Information

Supporting Table S1: Summarizes the detailed information of all eligible single nucleotide polymorphisms (SNPs) included in the bidirectional Mendelian randomization analysis, including SNP ID, effect allele, other allele, beta value of exposure and outcome, effect allele frequency, chromosome position, standard error, sample size, and *p* value. These SNPs were strictly screened according to genome‐wide significance threshold (*p* < 5 × 10^−8^), linkage disequilibrium criteria (kb = 10,000, *r* ^2^ = 0.001), and palindromic SNP exclusion rules to ensure the validity and reliability of instrumental variables in the causal inference between fasting blood glucose and myocardial infarction.

## Supporting information


**Supporting Information** Additional supporting information can be found online in the Supporting Information section.

## Data Availability

The data that support the findings of this study are available in https://wwwn.cdc.gov/nchs/nhanes/Default.aspx at https://wwwn.cdc.gov/nchs/nhanes/Default.aspx. These data were derived from the following resources available in the public domain: NHANES, https://wwwn.cdc.gov/nchs/nhanes/Default.aspx.
