# Supplementary material for: Association Between Fasting Blood Glucose and Myocardial Infarction Risk: Findings From the 2015–2018 NHANES Database and Mendelian Randomization Studies
Source: Cardiol Res Pract. 2026 Apr 20;2026:6984101. doi: 10.1155/crp/6984101 (PMC13093073; doi:10.1155/crp/6984101)
Supplement: Supplementary file 1 — Supporting Information Additional supporting information can be found online in the Supporting Information section. [file CRP-2026-6984101-s001.docx]

Table S1 | Mendelian Randomization Analysis Results of Fasting Glucose and Myocardial Infarction

| Exposure Factor | Outcome Factor | Analysis Method | Number of SNPs (n) | Odds Ratio (OR) | 95% Confidence Interval (95% CI) | P Value |
| --- | --- | --- | --- | --- | --- | --- |
| Fasting glucose | Myocardial infarction | MR-Egger | 68 | 1.0033 | (0.9991, 1.0074) | 0.1254 |
| Fasting glucose | Myocardial infarction | Weighted Median | 68 | 1.0032 | (1.0002, 1.0062) | 0.0387 |
| Fasting glucose | Myocardial infarction | Inverse Variance Weighted (IVW) | 68 | 1.0026 | (1.0006, 1.0046) | 0.0098 |
| Fasting glucose | Myocardial infarction | Simple Mode | 68 | 1.0033 | (0.9976, 1.0090) | 0.2652 |
| Fasting glucose | Myocardial infarction | Weighted Mode | 68 | 1.0031 | (1.0000, 1.0062) | 0.0506 |
| Myocardial infarction | Fasting glucose | MR-Egger | 66 | 0.9964 | (0.9747, 1.0187) | 0.7512 |
| Myocardial infarction | Fasting glucose | Weighted Median | 66 | 0.9923 | (0.9798, 1.0051) | 0.2377 |
| Myocardial infarction | Fasting glucose | Inverse Variance Weighted (IVW) | 66 | 0.9908 | (0.9821, 0.9996) | 0.0416 |
| Myocardial infarction | Fasting glucose | Simple Mode | 66 | 1.0036 | (0.9717, 1.0366) | 0.8288 |
| Myocardial infarction | Fasting glucose | Weighted Mode | 66 | 1.0061 | (0.9760, 1.0372) | 0.6496 |

Mendelian randomization analysis used a two-way design to explore the effect of fasting blood glucose on myocardial infarction and the reverse effect of myocardial infarction on fasting blood glucose. The results show that :

With fasting blood glucose as the exposure factor and myocardial infarction as the outcome, the IVW method ( core analysis method ) confirmed that there was a significant positive causal relationship between the two ( OR = 1.0026,95 % CI = 1.0006-1.0046, P = 0.0098 ). The results of the weighted median method were consistent and significant ( OR = 1.0032,95 % CI = 1.0002-1.0062, P = 0.0387 ). Although the P values of MR-Egger, simple mode and weighted mode did not reach a significant level, the effect direction was consistent with the core results. It is suggested that elevated fasting blood glucose will significantly increase the risk of myocardial infarction ;

in the reverse analysis, when myocardial infarction was the exposure factor and fasting blood glucose was the outcome, the IVW method showed that myocardial infarction had a significant effect on fasting blood glucose ( OR = 0.9908,95 % CI = 0.9821-0.9996, P = 0.0416 ), and the other methods had the same effect direction but did not reach a significant level, suggesting that there may be a two-way causal relationship between the two, but the effect intensity is weak ;

the heterogeneity test results showed that the Cochran Q test P values of all analyses were > 0.05 ( Table 1 ), indicating that there was no significant heterogeneity ; the P values of the MR-Egger intercept test were all > 0.05 ( Table 1 ), suggesting that there was no significant level of pleiotropy, and the results were robust and reliable.
